# Genetic Induction of the Warburg Effect Inhibits Tumor Growth

**DOI:** 10.18632/oncotarget.739

**Published:** 2012-11-14

**Authors:** Federica Sotgia, Ubaldo E. Martinez-Outschoorn, Michael P. Lisanti

**Affiliations:** Manchester Breast Centre & Breakthrough Breast Cancer Research Unit, Paterson Institute for Cancer Research; Institute of Cancer Sciences, Manchester Academic Health Science Centre, University of Manchester, UK; Department of Medical Oncology, Kimmel Cancer Center, Thomas Jefferson University, Philadelphia, PA; Manchester Breast Centre & Breakthrough Breast Cancer Research Unit, Paterson Institute for Cancer Research; Institute of Cancer Sciences, Manchester Academic Health Science Centre, University of Manchester, UK

Mitochondrial carriers are integral proteins of the inner membrane that allow for the exchange of metabolites, nucleotides, and cofactors between the cytosol and the mitochondria and thus, enact a variety of energetic adjustments [1,2].

The mitochondrial citrate carrier, SLC25A1 or CIC, catalyses the efflux of citrate from the mitochondrial matrix in exchange for cytosolic malate. Mitochondrial citrate is necessary for the Krebs cycle and oxidative phosphorylation (OXOPHOS), while cytosolic citrate provides the only carbon source for fatty acids and sterol biosynthesis. In addition, cytoplasmic citrate is an allosteric inhibitor of enzymes involved in glucose catabolism, particularly of phosphofructokinase (PFK), while at the same time providing a source for the production of NAD+ (via the action of citrate lyase and malate dehydrogenase), which can be used to support glycolysis. Because tumor cells display enhanced glycolytic capacity and enhanced rates of *de novo* lipogenesis, these activities would theoretically place CIC at a nodal point in the regulation of metabolic pathways and mitochondrial activity in cancer.

In this issue of *Oncotarget*, Avantaggiati and colleagues [3] provide novel findings that suggest that inhibition of tumor growth brought about by either genetic or biochemical inhibition of CIC occurs through unanticipated and fundamentally important new mechanisms that affect both cellular metabolism and viability. The authors show that while the inhibition of CIC blunted *de novo* lipid synthesis as expected, CIC inhibition also resulted in destabilization of the mitochondrial membrane potential, enhanced ROS (reactive oxygen species) production and increased production of L-lactate, indicative of a re-wiring of metabolism towards glycolysis.

The major implications of their findings is that a key component of CIC's ability to support proliferation of tumor cells might be in fact be the preservation of mitochondrial pathways of energy production while limiting the glycolytic addiction of tumor cells, essentially suppressing the Warburg effect, a metabolic trait that is proposed to promote malignancy. In addition, the authors provide compelling new evidence that CIC inhibition results in mitochondrial depletion and degradation *via* autophagy. In support of these data, blocking of autophagy rescues the anti-proliferative effects due to CIC loss both in tumor cells and in the model organism *Zebrafish*.

These studies describe a previously unknown role for CIC in regulating mitochondrial homeostasis and autophagy/mitophagy and are important and provocative for several reasons.

The Warburg theory proposes that the mitochondria of cancer cells are unable to provide energy via oxidative phosphorylation, and therefore rely upon glycolysis for energy production [4]. This view has been embraced in light of the fact that many oncogenic pathways and proteins, including Myc and Ras re-program the metabolism of cancer cells towards the glycolytic addiction, while inhibiting the expression of genes that promote mitochondrial oxidative phosphorylation (OXOPH). Conversely, various tumor suppressors including p53, inhibit glycolysis and promote mitochondrial respiration. These opposing activities support the view that an important metabolic trait of tumor cells is enhanced glycolysis and decreased mitochondrial respiratory capacity.

However, numerous lines of evidence fully support the existence of a much more complex interplay between the metabolism of glucose and mitochondrial activity, which is dependent upon the region of the tumor, -whether hypoxic or non-hypoxic- as well as the tumor microenvironment. For example, recent studies have provided for a model whereby hypoxic cancer cells convert glucose to lactate leading to its extracellular accumulation, where aerobic non-hypoxic cancer cells up-take it via monocarboxylate transporter 1 (MCT1) and utilize it for oxidative phosphorylation [5].

In the newly proposed “Two-Compartment Tumor Metabolism” model and the “Reverse Warburg Effect”, ROS produced by the tumor cells, activates autophagy and leads to mitochondrial dysfunction and glycolysis in cancer associated fibroblasts, which in turn results in the production of recycled nutrients, including L-lactate and ketone bodies, that drive the anabolic growth of tumors [6-9]. This feedback loop was shown to produce autophagy resistance and oxidative mitochondrial metabolism (OXPHOS) in cancer cells.

Thus, in this model of metabolic-coupling, the oxidative capacity of the tumor mitochondria and the concomitant bypassing of autophagy both act in concert to support cancer growth. Therefore, it is not unlikely that CIC is also involved in the positive-feedback and crosstalk provided by the stroma.

It will be important to determine whether CIC impinges upon other tumor-promoting metabolic branches, particularly the catabolism of glutamine.

## References

[1] Palmieri F, Pierri CL (2010). Essays Biochem.

[2] Sun J (2010). Mol Cell Pharmacol.

[3] Catalina-Rodriguez O (2012). Oncotarget.

[4] Koppeno WH (2011). Nat Rev Cancer.

[5] Sonveaux P (2008). J Clin Invest.

[6] Martinez-Outschoorn UE (2012). Cell Metab.

[7] Martinez-Outschoorn UE (2011). Cell Cycle.

[8] Sotgia F (2011). Breast Cancer Res.

[9] Fiaschi T (2012). Cancer Res.

